# Sodium butyrate and panobinostat induce apoptosis of chronic myeloid leukemia cells via multiple pathways

**DOI:** 10.1002/mgg3.613

**Published:** 2019-03-19

**Authors:** Xiaoyuan Jia, Yinsuo Zheng, Yanzi Guo, Kan Chen

**Affiliations:** ^1^ College of Life Sciences Zhejiang Sci‐Tech University Hangzhou China; ^2^ Department of Hematology Baoji Central Hospital Baoji China; ^3^ The Second Affiliated Hospital of Shaanxi Traditional University Xianyang China

**Keywords:** AKT‐mTOR pathway, apoptosis, chronic myeloid leukemia (CML), drug resistance, histone deacetylase inhibitors (HDACIs)

## Abstract

**Purpose:**

Histone deacetylase inhibitor (HDACI) is a novel therapeutic option for cancer. However, the effects of HDACIs on chronic myeloid leukemia (CML) and the underlying mechanisms are still unknown. The aim of this study was to investigate the effect and the mechanism‐of‐action of two HDACI members, sodium butyrate (NaBu) and panobinostat (LBH589) in K562 and the adriamycin–resistant cell line K562/ADR.

**Methods:**

Cell viability was assessed using MTT assay. Cell apoptosis was detected with flow cytometry. Cell cycle analysis and western blot were performed to explore the possible molecules related to HDACIs effects.

**Results:**

The effect of NaBu was more powerful on K562/ADR than on K562 cells. LBH589 triggered apoptosis and inhibited the growth of K562 cells. Both HDACIs inhibited K562 and K562/ADR cells via activation of intrinsic/extrinsic apoptotic pathways and inhibition of AKT‐mTOR pathway while NaBu also activated endoplasmic reticulum stress (ERS) mediated apoptotic pathway in K562/ADR cells. LBH589 reduced the expression of drug–resistant related proteins in K562 cells. However, neither NaBu nor LBH589 could significantly influence the expression of the drug–resistant related proteins in K562/ADR cells.

**Conclusion:**

The combination of HDACI and other therapeutic strategies are likely required to overcome drug resistance in CML therapy.

## 1 INTRODUCTION

Carcinogenesis involves not only genetic alterations but also epigenetic reprogramming, such as histone modification, DNA methylation and noncoding RNA deregulation. Among them, histone modification has been reported to associate with various types of cancers (Eckschlager, Plch, Stiborova, & Hrabeta, 2017; Thurn, Thomas, Moore, & Munster, 2011). Histone modification is regulated by histone acetyltransferase (HATs) or histone deacetylases (HDACs), adding or removing an acetyl group on ε‐amino lysine residues in histone N‐terminal region (Seidel, Schnekenburger, Dicato, & Diederich, 2012), and then activating transcription by nucleosome remodeling or transcription repression (Li et al., 2010; Monneret, 2005). Histone modification influences not only gene transcription (includes up‐regulation of antioncogenes and DNA repair genes) (Perri et al., 2017), but also changes the regulation of nonhistone protein (includes cellcycle, cell apoptosis and cell differentiation) (Mai et al., 2005). Recent studies have shown that the expression of HDACs is increased in both hematological cancers and solid tumors, which is associated with poor prognosis (Li et al., 2017; Stojanovic et al., 2017; Zhang et al., 2005). Thus, HDAC has emerged as a novel therapeutic target for cancer.

HDAC inhibitors (HDACIs) can interfere with the acetylation of histones in tumor cells and exert much lower toxicity on normal cells (Marks & Xu, 2009; Minucci & Pelicci, 2006). The antitumor effects of HDACIs have been confirmed in a variety of cancer cell lines, in vivo hematologic and solid tumors models (Benedetti, Conte, & Altucci, 2015; Crisanti et al., 2009). Several clinical trials have indicated beneficial effects in patients (Lee, Choy, Ngo, Foster, & Marks, 2010; Libby et al., 2015).

On the other hand, resistance to anticancer drugs is a major factor resulting in the failure of chemotherapy. Cancer drug resistance is characterized by multi–drug resistance (MDR), a phenomenon whereby cancers resistant to one drug are found to be resistant to other drugs with quite different structures and action modes (Higgins, 2007). Nowadays, resistance to HDACIs commonly occurs in clinic, nevertheless, not much is known about it.

In this study, we compared the anticancer effects of two HDACIs, sodium butyrate (NaBu), a short–chain fatty acid member of HDACIs, and panobinostat (LBH589), a cinnamic hydroxamic acid analog (class II HDACIs), in human chronic myeloid leukemia (CML) cell lines. LBH589 exhibited a stronger cell–killing effect than NaBu on K562 cells. NaBu was more efficient than LBH589 on K562/ADR (adriamycin–resistant K562) cells. Further investigation suggested that intrinsic, extrinsic, endoplasmic reticulum stress (ERS)–mediated apoptotic pathways and AKT‐mTOR pathway might be the underlying mechanisms for HDACIs related cell death. In addition, the expression of drug–resistant related proteins has been evaluated after the treatment of the two HDACIs.

## 2 MATERIAL AND METHODS

### 2.1 Cell culture and reagents

Human CML cell line K562 was obtained from the American Type Culture Collection (ATCC, Rockville, MD). K562/ADR, an adriamycin–resistant cell line, and K562/G, an imatinib–resistant cell line, were kindly provided by Dr. Wei He (Institute of Hematology, The First Affiliated Hospital, College of Medicine, Zhejiang University, China). All cells were maintained in RPMI‐1640 medium (Hyclone Laboratories) supplemented with 10% defined FBS (Hyclone Laboratories). Cells were cultured at 37°C in 5% CO_2_. Panobinostat (LBH589) was purchased from Selleck Chemicals (Houston, TX). Sodium butyrate (NaBu) was purchased from Sigma‐Aldrich (St. Louis, MO). The primary antibodies against β‐ACTIN, GAPDH, caspase‐3, caspase‐6, caspase‐8, caspase‐9, PARP, BIP, BCL‐XL, MCL‐1, BAX, BAK, BAD, XIAP, survivin, ABCG2, MDR1, BCR/ABL, p‐BCR/ABL, AKT, p‐AKT, mTOR, p‐mTORC1, 4EBP1, p‐4EBP1, eIF4E, p‐eIF4E and c‐MYC were purchased from Cell Signaling Technology (Danvers, MA), the antibody against caspase‐7 was purchased from Abcam (Cambridge, UK).

### 2.2 Cell viability assay

Cells were plated into 96‐well plates at 6,000 per well and treated with LBH589 and NaBu at serial concentrations (LBH589: 0, 25, 50, 100 and 200 nmol/L; NaBu: 0, 0.5, 1, 2, 4 and 8 mmol/L). At the culture time of 48 hr and 72 hr, 20 µl of 3‐(4, 5‐dimethylthiazol‐2‐yl)‐2, 5‐diphenyltetrazolium bromide (MTT) at a concentration of 5mg/ml was added into wells. Cells were incubated at 37°C for 4 hr, and the medium was removed immediately after centrifugation. Subsequently, 150 µl of DMSO was added and mixed thoroughly, absorbance was determined by using an enzyme mark instrument (Tecan DNA Expert) at 490 nm. Cell viability and IC_50 _values were analyzed by using GraphPad Prism V6.01 software.

### 2.3 Analysis of cell apoptosis

The apoptotic cells were measured as our previous studies (Chen et al., 2014). Cells (1 × 10^6^/6 mm dish) were plated into dishes. Two mmol/L of NaBu and 100 nmol/L of LBH589 were added respectively, and equal volumes of PBS buffer served as control. After 24 hr, cells were collected and resuspended in Annexin V‐binding buffer, and then stained with fluorescent–conjugated Annexin V and propidium iodide (PI) (FITC Annexin V Apoptosis Detection Kit; Becton Dickinson Biosciences) according to the manufacturer's instructions. Flow cytometric analysis was performed using FACS (Accuri C6; BD Biosciences).

### 2.4 Analysis of cell cycle

Cells (1 × 10^6^/well) were seeded into 6‐well plates followed by adding 2 mmol/L NaBu and 100 nmol/L LBH589 respectively. After 24 hr, the cells were collected and resuspended in 600 µl ice–cold PBS, and then fixed in ice–cold 70% ethanol overnight at −20°C. Subsequently, the cells were washed twice with PBS and incubated with 5 µl RNase (100 mg/ml) at 37°C for 30 min, and then incubated with 50 µg/ml propidium iodide (PI) at 4°C for 30 min. The samples were analyzed immediately using FACS. Cell cycles were analyzed by using FlowJo VX software.

### 2.5 Western blot analysis

Cells were harvested and lysed with 1 × cell lysis buffer (Cell Signaling Technology, Danvers, MA) containing 1 mmol/L PMSF at 4°C for 30–60 min. Samples of cytosolic proteins were formed by centrifugation at 12,000 *g* for 10 min. The concentrations of protein were measured using BCA method (Pierce® BCA Protein Assay Kit; Thermo Fisher Scientific, Inc., Rockford). Samples containing 20–50 μg total proteins were separated using 10%–12% SDS–PAGE gel and transferred onto PVDF membranes (Millipore, Bedford, MA). The membranes were blocked with 5% nonfat milk at room temperature for 2 hr and incubated with primary antibodies (1:1,000 dilutions) overnight at 4°C. Next day, the membranes were washed with TBS buffer containing 0.05% (v/v) Tween 20 (TBST) buffer and incubated with horseradish peroxidase (HPR)–conjugated secondary antibodies (1:5,000 dilution; Lianke Biotech, Co., Ltd. Hangzhou, China) at room temperature for 2 hr. After washing with TBST, the membranes were then visualized using ECL detecting kit (PerkinElmer, Inc., MA) and Tanon 5,500 gel imaging system (Tanon Science & Technology Co., Ltd. Shanghai, China).

## 3 RESULTS

### 3.1 HDACIs inhibited cell proliferation and induced cell apoptosis in K562 cells

**Figure 1:**
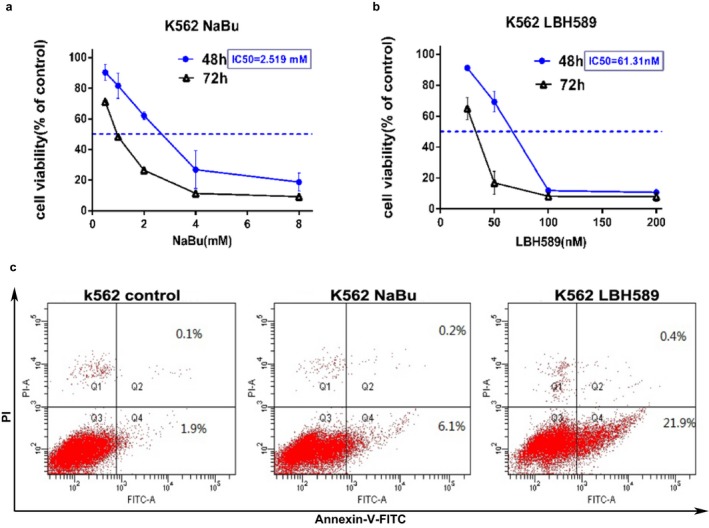
HDACIs inhibited cell proliferation and induced cell apoptosis of K562 cells. Cell survival rates were measured at 48 hr and 72 hr using the MTT assay after treatment with different concentrations of NaBu (a) and LBH589 (b). The results represent the mean of at least three independent experiments. Data are presented as mean ± *SD*. (c) The apoptosis of K562 cells was analyzed using flow cytometry after treatment of NaBu and LBH589. Cells were treated with 2 mmol/L NaBu or 100 nmol/L LBH589 for 24 hr, and apoptosis status was analyzed using Annexin V‐FITC and PI double staining. Early apoptotic cells are Annexin V^+^/PI^−^, and late apoptotic and dead cells are Annexin V^+^/PI^+^

To explore the effect of NaBu and Panobinostat on K562 cell line, the cells were treated with serial concentrations of NaBu and LBH589 for 24, 48 and 72 hr respectively. MTT assays showed that the two HDACIs can inhibit the proliferation of K562 cells in a dose‐ and time‐dependent manner. The IC_50 _values of NaBu and LBH589 (48 hr) were 2.591 mmol/L and 61.31 nmol/L, respectively (Figure 1a–b). To evaluate the effect of cell apoptotic induction, flow cytometry was performed after the treatment of NaBu or LBH589. The results showed that LBH589 significantly induces cell apoptosis in K562 (Figure 1c).

### 3.2 HDACIs induced cell apoptosis by activating intrinsic and extrinsic apoptotic pathways in K562 cells

**Figure 2:**
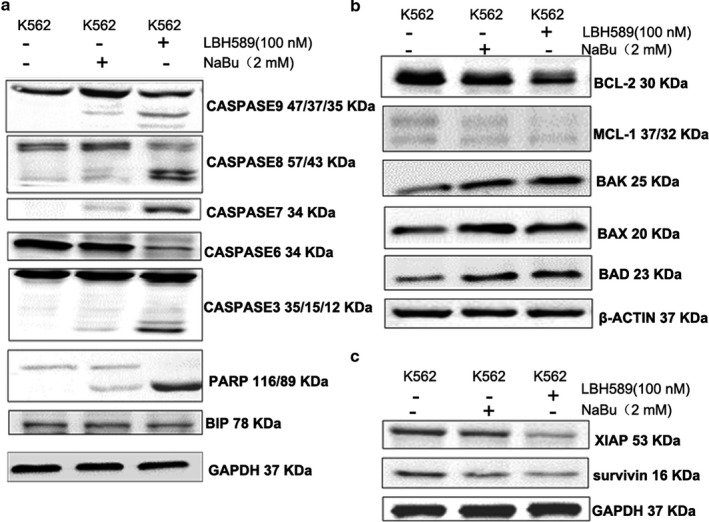
LBH589 activated the apoptosis related proteins in K562 cells. Cells were treated with 2 mmol/L NaBu or 100 nmol/L LBH589 for 24 hr. (a) The expressions of caspase 9/8/7/6/3 and PARP were significantly induced by the treatment of the two HDACIs. (b) The expressions of BCL‐2 family proteins were influenced by the treatment of the two HDACIs. (c) As the members of the inhibitor of apoptosis family of proteins (IAP), XIAP and survivin were down‐regulated by the treatment of HDACIs

To further investigate the apoptotic signaling pathways involved in the two HDACIs stimulus, the protein levels of caspase family, BCL‐2 family, an inhibitor of apoptosis protein (IAP) family, BIP and ERS–mediated apoptotic proteins were detected using western blot assay. The results showed that LBH589 apparently increases the cleavage of caspase‐9, ‐8, ‐7, ‐3 and PARP (Figure 2a), while reducing the expression of procase‐9, ‐8, ‐6, and ‐3. This demonstrated both the intrinsic and extrinsic apoptotic pathways are triggered by the two HDACIs. In addition, LBH589 significantly decreased the levels of BCL‐XL and MCL‐1, but increased the levels of BAK and BAX (Figure 2b).

To confirm the effect of HDACIs on the inhibitor of apoptosis protein (IAP) family, we detected the expression of XIAP and survivin at protein levels, which are the most well–characterized members of IAP family (Siddiqui, Ahad, & Ahsan, 2015). As expected, the expression of XIAP and survivin were inhibited by LBH589 (Figure 2c). NaBu had similar but weaker effects than LBH589 on the two proteins.

### 3.3 HDACIs induced K562 cell apoptosis by suppressing AKT‐mTOR pathway

**Figure 3:**
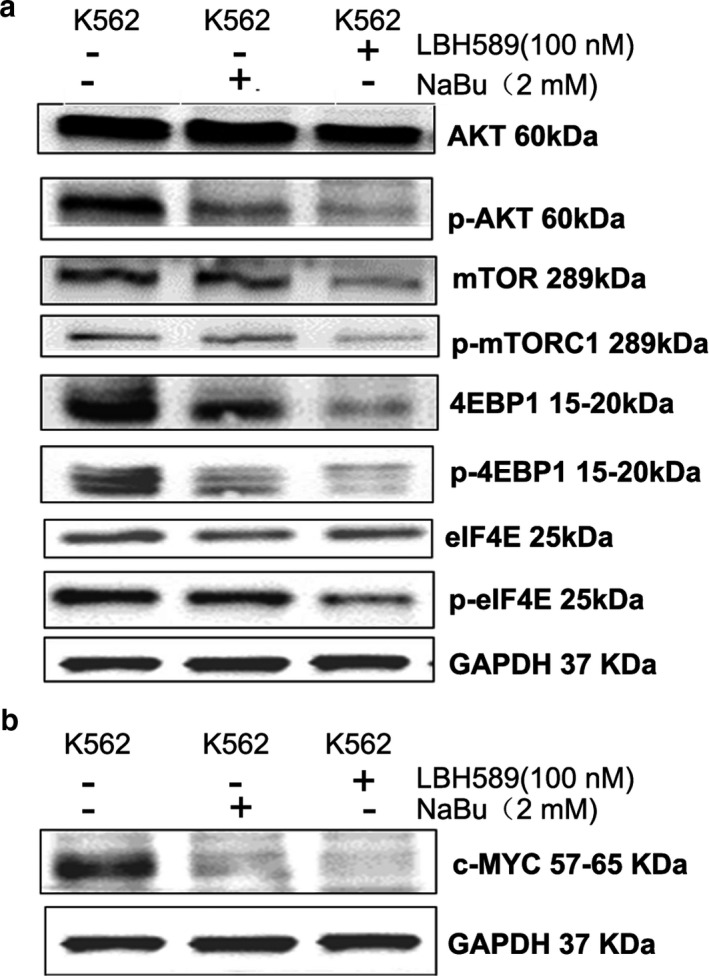
HDACIs inhibited the activity of AKT‐mTOR signaling pathway in K562 cells. Cells were treated with 2 mmol/L NaBu or 100 nmol/L LBH589 for 24 hr. (a) The expressions of key proteins in AKT‐mTOR signaling pathway were dramatically changed after treatment of HDACIs; (b) Transcription factor c‐MYC was down‐regulated by HDACIs treatment

Aberrant activation of the AKT‐mTOR (protein kinase B/mammalian target of rapamycin) cascade had been found in various cancers (Laplante, & Sabatini, 2012; Raphael, Desautels, Pritchard, Petkova, & Shah, 2018). Overexpression of downstream mTOR effectors, including eIF4E (eukaryotic translation initiation factor 4E), S6K and 4EBP1 (eIF4E‐binding protein 1) was reported to be associated with poor prognosis (Helena, Manuel, & Paula, 2012). To explore the influence of HDACI on the AKT‐mTOR signaling pathway, the key proteins in AKT‐mTOR pathway were determined after treatment of HDACIs in K562 cells. However, HDACIs remarkably diminished the levels of phosphorylated AKT protein while they did not obviously affect the total amount of AKT. In addition, the down‐regulation of mTOR (p‐mTORC1, 4EBP1 and p‐4EBP1) signaling was also observed after treatment of LBH589 or NaBu in K562 cells (Figure 3a). This suggested that the AKT‐mTOR signaling pathway acted as a target for HADCI.

Previous study reported that down‐regulation of transcription factor c‐MYC induces cell apoptosis via an AMPK/mTOR dependent pathway (Shin et al., 2016). In this study, the expression of c‐MYC was measured to investigate the relationship between c‐MYC and HDACI treatment. The result showed that both the HDACIs down‐regulate the expression of c‐MYC at protein level in K562 cells (Figure 3b).

### 3.4 NaBu provokes G0/G1–phase arrest in K562 cells

**Figure 4:**
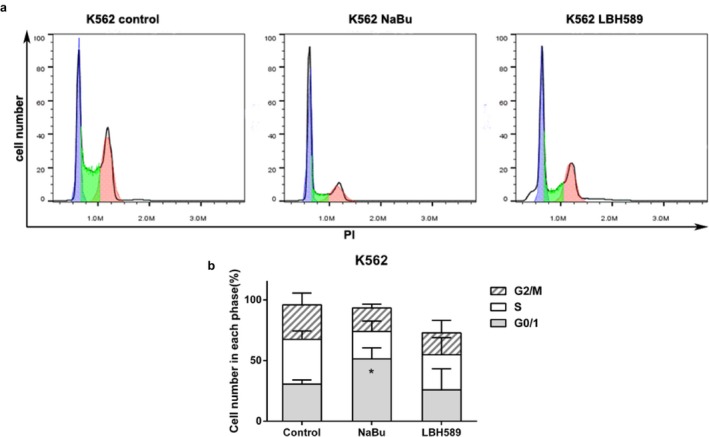
NaBu arrested cell cycle in G0/G1 phase. (a) The treatment of 2 mmol/L NaBu caused a significant increase in the G0/G1 fraction of the cell cycle in K562 cells, while 100 nmol/L LBH589 failed to perturb cell cycle obviously; (b) Quantification of cell cycle analysis. Data were presented as mean ± *SD*, *p < 0.05

To further investigate the effect of HDACIs on cell cycle progression, K562 cells were collected and cell cycle analysis was performed by using flow cytometry. The result showed that NaBu significantly arrests the cells in G0/G1‐phase (51.35% ± 9.12%) when compared with control (30.6% ± 3.39%). These results were in line with the previous study (Li et al., 2008). However, LBH589 failed to perturb the cell cycle in K562 cells (Figure 4a–b).

### 3.5 HDACIs failed to down‐regulate the expression of drug resistance–related proteins in K562/ADR cells

**Figure 5:**
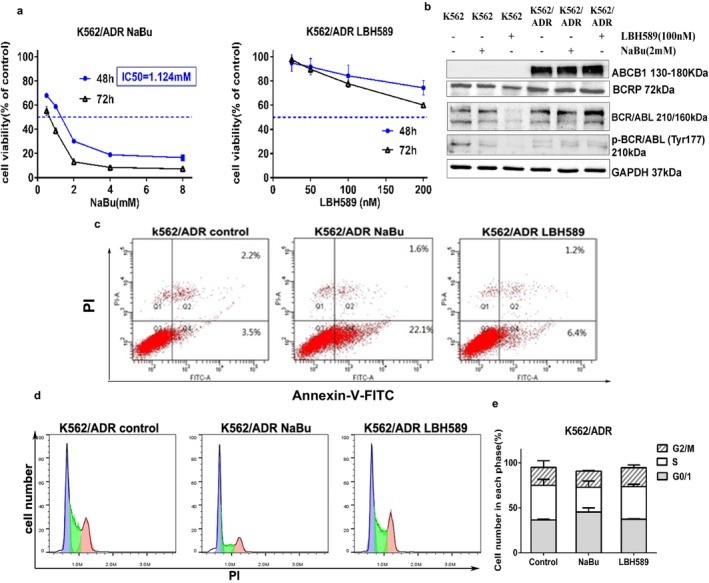
The effects of HDACIs on K562/ADR cells. Cells were treated with 2 mmol/L NaBu or 100 nmol/L LBH589 for 24 hr. (a) NaBu significantly inhibited the proliferation of K562/ADR cells while LBH589 had a moderate effect on the cells; (b) Neither NaBu nor LBH589 could suppress the expression of drug–resistance related proteins in K562/ADR cells, while LBH589 down‐regulated the expression of ABCG2, BCR/ABL and p‐BCR/ABL in K562 cells; (c) NaBu significantly induced apoptosis in K562/ADR cells; (d and e) Neither of the HDACIs exhibited cell cycle arrest in K562/ADR cells

Since the two HDACIs, especially LBH589 had been demonstrated to exert cytotoxicity in K562 cells. It is necessary to further explore the effects of the two HDACIs on drug–resistant cells. K562/ADR (an adriamycin–resistant cell line) and K562/G (an imatinib–resistant cell line) were used as experimental models in this part. Our results showed that NaBu inhibited the proliferation of K562/ADR and K562/G cells in a dose‐ and time‐dependent manner. The IC_50 _values are 1.124 mmol/L for K562/ADR and 1.108 mmol/L for K562/G. However, the MTT assay demonstrated that LBH589 possesses potent inhibitory effect on K562/G cells and moderates an inhibitory effect on K562/ADR (Figure S1a and Figure 5a). NaBu exhibited a more dramatic cytotoxic effect than LBH589 on K562/ADR cells (Figure S1 and Figure 5a).

We next focused on K562/ADR cells and investigated the drug–resistance mechanisms of the two HDACIs. It was reported that the expression of ABCB1 (also known as P‐glycoprotein or MDR1) and breast cancer resistance protein (BCRP, also known as ABCG2) is elevated **i**n drug–resistant leukemia cell lines (Baran et al., 2007; Ho, Hogge, & Ling, 2008). Some studies suggested that MRP (multi–drug resistance protein) family members(Grant et al., 1994; Legrand, Simonin, Zittoun, & Marie, 1998; Zaman et al., 1994), SIRT1 (silent information regulator 1), (Chu, Chou, Zheng, Mirkin, & Rebbaa, 2005; Li et al., 2014; Wang & Chen, 2013) and LRP (lung resistance–related protein) (Scheffer et al., 1995; Valera, Scrideli, Queiroz, Mori, & Tone, 2004) are also involved in the multi–drug resistant progress, Meanwhile, the formation of BCR‐ABL protein was demonstrated conferring resistance to multiple genotoxic anticancer agents (Bedi et al., 1995; Kantarjian, Giles, Quintáscardama, & Cortes, 2007; Yasufumi et al., 2016) and was considered to be the cause of CML (Maru, 2012). Thus, BCR‐ABL remains a therapeutic target in all phases of CML (Fiskus et al., 2006). In our study, mRNA levels of P‐glycoprotein, BCRP, ABCB5, MRP1 (multi–drug resistance protein1, also known as ABCC1), MRP2 (multi–drug resistance protein2, also known as ABCC2), MRP3 (multi–drug resistance protein3, also known as ABCC3), SIRT1 and LRP were measured after treatment of NaBu or LBH589. Meanwhile the protein levels of ABCB1, BCRP, BCR/ABL and p‐BCR/ABL were also detected post NaBu or LBH589 treatment.

Treated with LBH589 or NaBu, the mRNA levels of those drug resistance–related proteins were upregulated (Figure S2). As shown in Figure 5b, the expressions of ABCB1 and BCRP were abundant in K562/ADR cells. Neither LBH589 nor NaBu decreased the expression of these two proteins in K562/ADR cells. Interestingly, LBH589 remarkably down‐regulated the expression of BCRP, BCR/ABL and p‐BCR/ABL in K562 cell, which maybe explain the powerful cell–killing effect of LBH589 on K562 cells.

### 3.6 NaBu induced apoptosis of K562/ADR cells via intrinsic, extrinsic, ERS–mediated pathways and AKT‐mTOR pathway

To determine the proapoptotic activities of NaBu and LBH589 in K562/ADR cells, Annexin V‐FITC/PI double staining was performed. The results showed that NaBu induces more cell apoptosis than LBH589 (Figure 5c). Our cell cycle analysis indicated that neither of the two HDACIs apparently induces cell cycle arrest in K562/ADR cells (Figure 5d and e).

**Figure 6:**
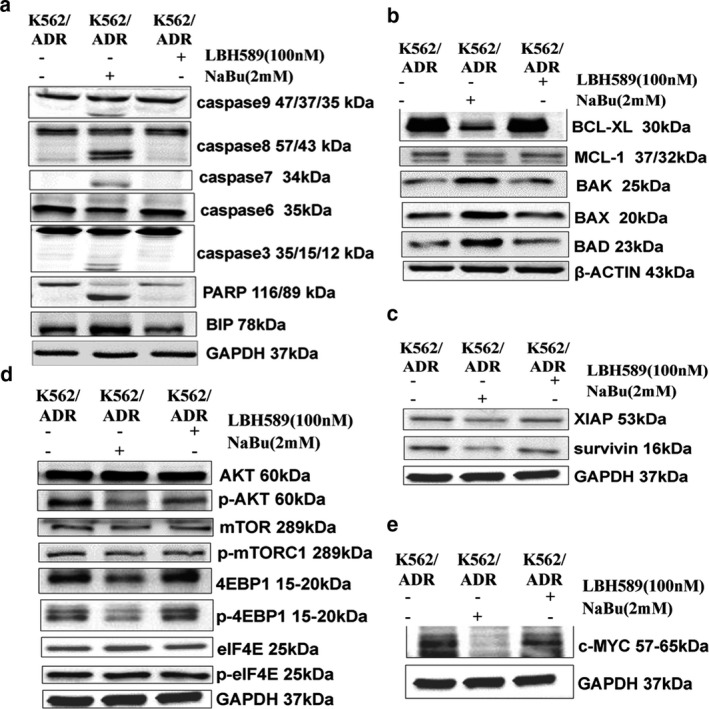
NaBu induced K562/ADR cells apoptosis via intrinsic, extrinsic, ERS–mediated pathways and AKT‐mTOR pathway. (a, b and c) The effects of HDACIs on apoptotic related proteins in K562/ADR cells; (d and e) NaBu inhibited the activity of AKT‐mTOR pathway and down‐regulated the expression of c‐MYC in K562/ADR cells

To investigate the underlying molecular mechanism of NaBu inducing apoptosis, we detected the levels of proteins involved in the apoptotic pathways and AKT‐mTOR pathway using western blot assay. The results suggested that NaBu, but not LBH589, activated all of the intrinsic, extrinsic and ERS–mediated apoptotic pathways (Figure 6a–c) in K562/ADR cells. Subsequently, NaBu could also suppress the activity of AKT‐mTOR signaling and its downstream target, c‐MYC (Figure 6d–e).

## 4 DISCUSSION

Since HDACs are dysregulated in many cancers, a variety of HDACIs have been investigated in respect to their anticancer effects in various experimental cancer models and clinical studies (Eckschlager et al., 2017; Marks & Xu, 2009; Ceccacci & Minucci, 2016). HDACIs have been classified into five main classes according to their specificity for HDAC subtypes: (1) Hydroxamic acids; (2) Short–chain fatty acids; (3) Benzamides; (4) Cyclic tetrapeptides; (5) Sirtuin inhibitors, including the pan‐inhibitor nicotinamide (Eckschlager et al., 2017). As a member of HDACIs, sodium butyrate (NaBu) has been reported to arrest cell cycle in the G0/G1 phase (Candido, Reeves, & Davie, 1978), trigger cell apoptosis (Hague et al., 1993; Pajak, Orzechowski, & Gajkowska, 2007) and induce cell differentiation (Rahman et al., 2003). As a novel Pan‐HADCI, LBH589 possesses a promising activity against hematologic and solid tumors (George et al., 2005; Prince, Bishton, & Johnstone, 2009).

In this study, we confirmed the antitumor effects of the two HDACIs, NaBu and LBH589, on K562 and K562/ADR cell lines. Interestingly, the cell–killing effect of LBH589 on K562 cells was more distinct than that on K562/ADR cells. LBH589 exerted a minor effect on drug–resistance related proteins, which may explain the limitation of LBH589 in anticancer therapy. On the other hand, NaBu exhibited stronger cytotoxicity in K562/ADR cells than in K562 cells, which suggests that NaBu might be a more potent drug for multi–drug resistance.

Cell apoptosis is a highly regulated process and two major apoptotic pathways, intrinsic (mitochondrial) and extrinsic (death receptor) pathways, are involved. In these two pathways, initiator caspases (caspase‐8, ‐9 etc.) are active and induce executioner caspases (caspase‐3, ‐6, ‐7), then proteins are degraded and the cells undergoapoptosis. Poly (ADP‐ribose) polymerase (PARP) is the main target of caspase‐3*,* and cleavage PARP serves as a marker of cells undergoing apoptosis (Oliver et al., 1998). To examine the main apoptotic pathway in HDACIs treatment, the expression of the key proteins in these two pathways were detected. As shown in our results, both of the intrinsic and extrinsic pathways were activated by LBH589 and NaBu. As ERS–mediated apoptosis was proved to be the third progress (Pfaffenbach & Lee, 2011), we also measured the expression of ERS–related protein. The results showed that BIP significantly increases after NaBu treatment in K562/ADR cells, thus suggested that ERS–mediated apoptotic progress is involved in NaBu induction.

The BCL‐2 family regulates mitochondrial permeability and plays a role in the progression of apoptosis. All BCL‐2 family members can be divided into proapoptotic proteins (e.g. BAX, BAK, BIM, BID and BAD) and antiapoptotic proteins (eg. BCL‐2, BCL‐XL, and MCL‐1). The ratio of pro and antiapoptotic proteins determines the sensitivity of the cells to apoptotic stimulus (Siddiqui et al., 2015).

Multi–drug resistance is the main obstacle in cancer therapy. ABCB1, MRPs and BCRP are efflux transporters involved in multi–drug resistance in cancer cells (Ji et al., 2009; Mao & Unadkat, 2015; Sodani, Patel, Kathawala, & Chen, 2012). Previous studies reported that ABCB1 is expressed in K562/ADR cells (Kato, Ideguchi, Muta, Nishimura, & Nawata, 1990), and the up‐regulation of MCL‐1 protein induces multi–drug resistance to doxorubicin and other standard therapies in leukemia (Hermanson, Das, Li, & Xing, 2013; Ji et al., 2009). Thus, targeting both ABCB1 and MCL‐1 may help overcome drug resistance in human leukemia (Ji et al., 2009). In our study, K562/ADR cells express higher levels of MCL‐1 and ABCB1. However, HDACIs treatment failed to decrease the level of the drug–resistant related proteins though NaBu efficiently induced K562/ADR cells apoptosis. Recently, it has been reported that an oral multi–targeted tyrosine kinase inhibitor (TKI) can strongly reverse MDR and enhance the efficacy of drugs in K562/ADR cells without altering the expression of ABCB1 at both mRNA and protein levels (Tong et al., 2012). Therefore, NaBu might be a potent drug for cancer therapy independent of the ABC protein superfamily.

The mammalian target of rapamycin (mTOR) pathway is abnormally activated in various cancers and thus plays significant roles in cancer cell survival and growth. The mTORC1 is one form of the mTOR complex, which is the downstream target of phosphatidylinositol 3 kinase (PI3K)/AKT signaling pathway. It can phosphorylate and inactivate 4EBP1, and then release eIF4E to trigger mRNA translation. As a key element of the initiation translation complex, eIF4E participates in the translation of tumor–associated proteins, such as c‐MYC, cyclin D1, and MCL‐1 (Kosciuczuk, Saleiro, & Platanias, 2017). The phosphorylation of eIF4E can enhance and facilitate the nucleo–cytoplasmic transport of certain mRNAs and then influence cell cycle and cell survive (Kosciuczuk et al., 2017; Topisirovic, Ruizgutierrez, & Borden, 2004). Our results showed that LBH589 down‐regulated the level of phosphorylated eIF4E in K562 cells, which indicated the strong effect of LBH589 in K562 cells. Several studies have reported that HDACIs can block AKT/mTOR signaling in many cancers (Kawamata, Chen, & Koeffler, 2007; Ou et al., 2015; P. Zhang et al., 2015), while the relationship between HDACI induction and AKT/mTOR pathway in CML cell remains unclear. Our results showed that HDACIs remarkably diminish the level of phosphorylated AKT protein without obvious effect on the total amount of AKT. Similarly, the down‐regulation of mTOR, p‐mTORC1, 4EBP1, and p‐4EBP1 was observed after HDACIs treatment, which is consistent with our cell apoptosis results. In addition, we found that HDACIs treatment obviously decreases the expression of c‐MYC in K562 cells, which confirmed the previous study (Shin et al., 2016) that the activation of AKT confers resistance to chemotherapy. Thus, the inhibition of AKT might be a valid approach for cancer treatment (West, Castillo, & Dennis, 2002). In this study, we propose that the two HDACIs possess the capability of inducing CML cell apoptosis, which is involved in AKT/mTOR signaling suppression.

## 5 CONCLUSION

In summary, the two HDACIs exhibited distinct cell–killing effects on CML cells. Both of them were involved in the intrinsic, extrinsic apoptotic progress and AKT‐mTOR pathway. NaBu inhibited the growth of drug resistance CML cells independent on MDR signaling. Further exploration is needed to figure out the mechanism‐of‐action. Considering the limitation of LBH589 in drug resistance, combination with other therapeutic drugs is likely required in clinical cancer therapy.

## DISCLOSURE

The authors report no conflict of interest in this work.

## AUTHOR CONTRIBUTIONS

X.J. performed the experiments and data analysis; X.J. and Y.Z. were responsible for acquisition of data, analysis, and interpretation of data and drafting of the manuscript; Y.G. was involved in project discussion and data analysis/interpretation; K.C. was responsible for study design, analysis, and revision of the manuscript.
