# Supplementary material for: Sodium butyrate and panobinostat induce apoptosis of chronic myeloid leukemia cells via multiple pathways
Source: Mol Genet Genomic Med. 2019 Mar 19;7(5):e613. doi: 10.1002/mgg3.613 (PMC6503025; doi:10.1002/mgg3.613)
Supplement: Supplementary file 1 [file MGG3-7-e613-s001.doc]

**Supplementary information**

Supplementary figure S1


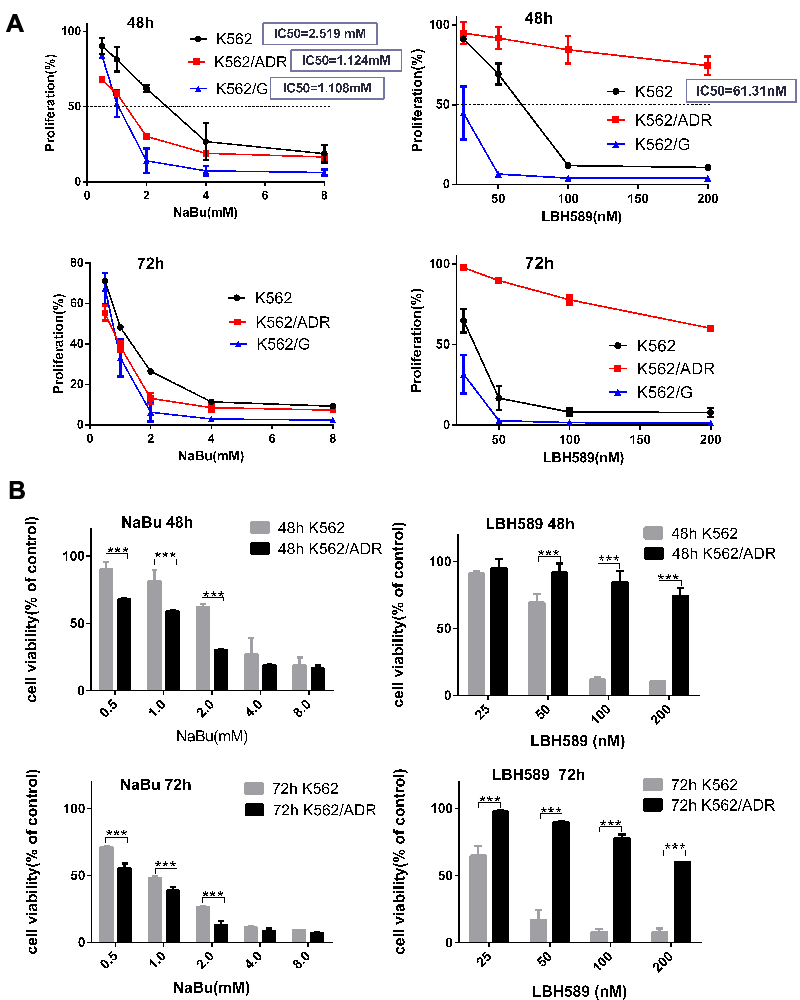


**Figure S1. HDACIs inhibited cell proliferation in K562 and K562/ADR cells after treatment of 48 h and 72 h.** (A) Cell proliferation was measured in 48 h and 72 h after HDACIs treatment at different concentrations. (B) Cell viability was measured after HDACIs treatment. The results represented the mean of at least three independent experiments, data were presented as mean ± SD. ***P < 0.001.

Supplementary figure S2


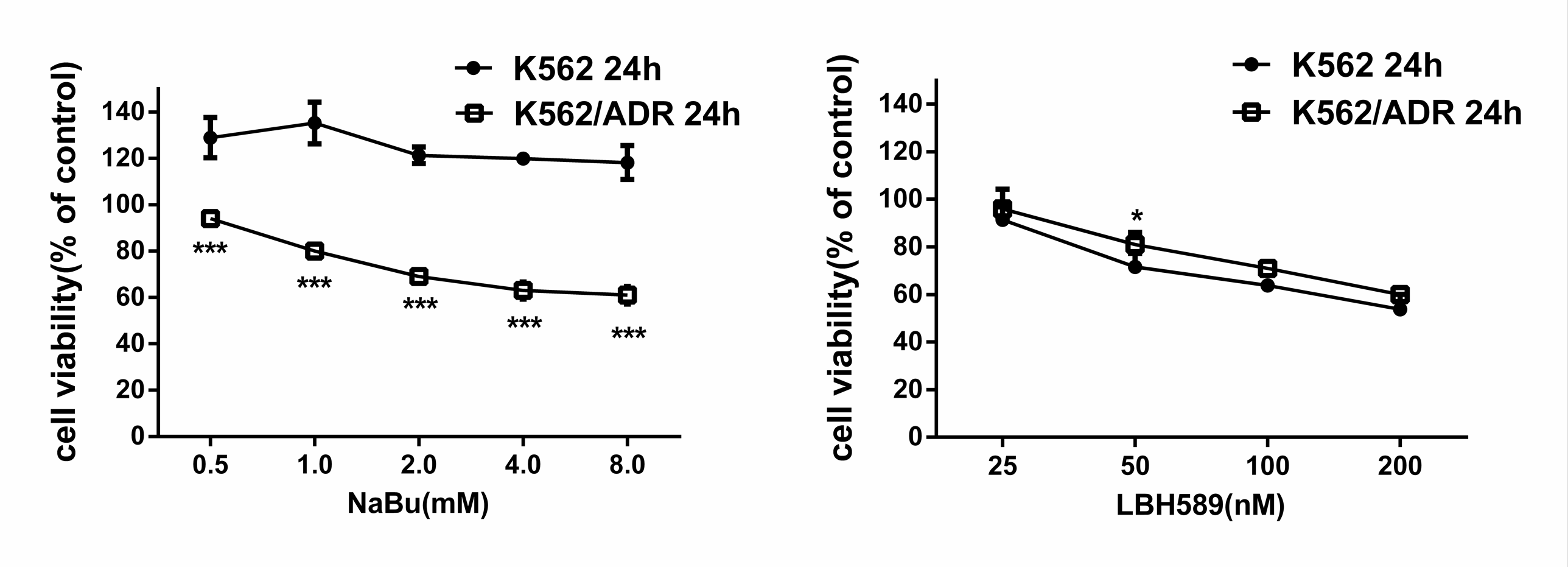


**Figure S2. HDACIs inhibited cell proliferation in K562 and K562/ADR cells after treatment of 24 h.** Cell viability was measured in 24 h after HDACIs treatment at different concentrations. The results represent the mean of at least three independent experiments, data were presented as mean ± SD. *P < 0.05, *******P < 0.001.

Quantitative real-time PCR

Methods:

Total RNA was isolated from chronic myeloid leukemia cells with RNA-Quick Purification Kit (ES Science, China) and the complementary DNA was synthesized by qPCR RT Kit (Toyobo, Osaka, Japan) according to the manufacturer’s instructions. Quantitative real-time PCR was performed using SYBR Green kit (Toyobo, Osaka, Japan). Real-time PCR was carried out by the ABI Prism 7500 Sequence Detector (Applied Biosystems, Foster City, CA, USA). The ΔΔCt method with relative quantification of gene expression was used to determine mRNA levels. The housekeeping gene, glyceraldehyde-3-phosphate dehydrogenase (GAPDH), was used for normalization. The primer sequences of MRP1, MRP2, MRP3, ABCB1, ABCB5, SIRT1, BCRP, LRP and GAPDH are shown in Table S1.

**Table S1 The primers used in Q-PCR assay**

| **Primer** | **Forward primer** | **Reverse primer** |
| --- | --- | --- |
| MRP1 | TCTCTCCCGACATGACCGAGG | CCAGGAATATGCCCGACTTC |
| MRP2 | ACGGACAGCTATCATGCTTCT | TGGTCACACCATGAGCTTCT |
| MRP3 | CTTAAGACTTCCCCTCAACATGC | GGTCAAGTTCCTCTTGGCTCA |
| ABCB1 | CAGGACATAGGCTGGTTTGATGGT | TTAGCTTCCAACCACGTGTAAATC |
| ABCB5 | TCACGCAGTCACCTTTCCTCTC | CTTGGTTGTCATCCAGCAGCT |
| SIRT1 | CGGAAACAATACCTCCACCTGA | GAAGTCTACAGCAAGGCGAGCA |
| BCRP | GGCCTCAGGAAGACTTATGT | ATCAGCTACACCACCTCCTT |
| LRP | GTCTTCGGGCCTGAGCTGGTGTCG | AAGGACCCCCAAGAGACGGCCAAG |
| GAPDH | TTCGGATCCACTCTTTCACCTT | TGGAAGATGGTGATGGGAT |





**Figure S3. Relative mRNA levels of MRP1, MRP2, MRP3, ABCB1, ABCB5, BCRP, SIRT1 and LRP in K562 and K562/ADR cells.** Relative mRNA levels were measured at 24 h by qPCR assay after treatment of 2mM NaBu or 100nM LBH589. GAPDH was used as normalization. Data were presented as mean ± SD, *P < 0.05, **P < 0.01, ***P < 0.001.
